# Replanning Criteria and Timing Definition for Parotid Protection-Based Adaptive Radiation Therapy in Nasopharyngeal Carcinoma

**DOI:** 10.1155/2015/476383

**Published:** 2015-12-17

**Authors:** Wei-Rong Yao, Shou-Ping Xu, Bo Liu, Xiu-Tang Cao, Gang Ren, Lei Du, Fu-Gen Zhou, Lin-Chun Feng, Bao-Lin Qu, Chuan-Bin Xie, Lin Ma

**Affiliations:** ^1^Department of Radiation Oncology, Chinese PLA General Hospital, 28 Fuxing Road, Beijing 100853, China; ^2^Department of Oncology, People's Hospital of Jiangxi Province, 92 Aiguo Road, Nanchang 330006, China; ^3^Image Processing Centre, Beihang University, 37 Xueyuan Road, Beijing 100191, China; ^4^Department of Medical Statistics, Chinese PLA General Hospital, 28 Fuxing Road, Beijing 100853, China; ^5^Department of Radiation Oncology, Air Force General Hospital, 30 Fucheng Road, Beijing 100141, China; ^6^Department of Radiation Oncology, Chinese PLA General Hospital, Hainan Branch, Haitang Bay, Sanya 572000, China

## Abstract

The goal of this study was to evaluate real-time volumetric and dosimetric changes of the parotid gland so as to determine replanning criteria and timing for parotid protection-based adaptive radiation therapy in nasopharyngeal carcinoma. Fifty NPC patients were treated with helical tomotherapy; volumetric and dosimetric (*D*_mean_, *V*_1_, and *D*_50_) changes of the parotid gland at the 1st, 6th, 11th, 16th, 21st, 26th, 31st, and 33rd fractions were evaluated. The clinical parameters affecting these changes were studied by analyses of variance methods for repeated measures. Factors influencing the actual parotid dose were analyzed by a multivariate logistic regression model. The cut-off values predicting parotid overdose were developed from receiver operating characteristic curves and judged by combining them with a diagnostic test consistency check. The median absolute value and percentage of parotid volume reduction were 19.51 cm^3^ and 35%, respectively. The interweekly parotid volume varied significantly (*p* < 0.05). The parotid *D*_mean_, *V*_1_, and *D*_50_ increased by 22.13%, 39.42%, and 48.45%, respectively. The actual parotid dose increased by an average of 11.38% at the end of radiation therapy. Initial parotid volume, initial parotid *D*_mean_, and weight loss rate are valuable indicators for parotid protection-based replanning.

## 1. Background

Due to the anatomical and biological specificity of nasopharyngeal carcinoma (NPC), radiation therapy or chemoradiotherapy has been recognized as a definitive treatment. Studies have shown that the higher the radiation dose delivered to the target volume, the better the local disease control ratio [1]. The escalation of the delivered dose, however, often leads to severe and related side-effects. Xerostomia is one of the most frequent side-effects and the amount of radiation that is delivered to the parotid glands, assuming a major role in stimulating salivary flow, affects NPC patients' quality of life. Therefore, when treating NPC it is crucial to minimize the dose to the parotid glands while ensuring adequate dose distribution to the target volumes. Unlike two-dimensional conventional radiation therapy (2DCRT) and three-dimensional conformal radiation therapy (3DCRT), intensity modulated radiation therapy (IMRT) can deliver a highly conformal dose to targets while effectively sparing critical normal organs, potentially improving the local control rate and reducing radiation-related toxicities [2–5].

Patients with head and neck cancer may be subjected to significant anatomical changes during radiation therapy, changes which can cause volume shrinkage near the facial surface [6–8]. And parotid gland variations may result in an unanticipated overdose [9, 10]. Replanning during radiation therapy can correct these anatomical changes and protect parotid glands from an overdose of irradiation [10–12]. However, the indications and timing of replanning are still unknown.

Helical tomotherapy (HT) is a unique IMRT modality that combines elements of diagnostic radiology and radiation therapy in a single unit. In addition to its ability to deliver a highly conformal dose distribution, HT is equipped with xenon detectors that have been designed to obtain the megavoltage computed tomography (MVCT) images that are used for pretreatment set-up verification and actual dose detection [13]. In the previous study, we evaluated weekly anatomical and dosimetric changes of the parotid gland in 35 NPC patients treated with HT system and found that some patients' parotid volumes and locations varied significantly, generally causing an increase of the actual delivered dose [14]. It is thus necessary to identify relevant factors that affect these changes. We performed this study in order to evaluate the real-time volumetric and dosimetric changes of the parotid gland and thus determine the replanning cut-off values for parotid protection-based adaptive radiation therapy (ART) in NPC.

## 2. Materials and Methods

### 2.1. Patients

We pooled data from the 100 parotid glands of 50 NPC patients that were treated with the TomoTherapy Hi-Art Treatment System (Accuray, Sunnyvale, CA). The patients were all initially diagnosed, histologically proven, and had a median age of 44 years (range: 11–80 years). Thirty of the 50 patients received platinum-based chemotherapy, and 20 others received only radiation therapy. Informed consent was obtained from all patients before receiving treatment. The main patient characteristics are summarized in Table 1.

### 2.2. Delineation and the Dose to Target Volumes and Organs at Risk (OARs)

The process of CT simulation and the delineation of targets and OARs have been previously reported [14]. Briefly, the gross target volume of the primary tumor (GTVnx) and metastatic lymph nodes (GTVnd) were, respectively, defined as the visible tumor and involved nodes. The pGTVnx was obtained by expanding the corresponding GTVnx by a margin of 3–5 mm and being limited by the brainstem, spinal cord, optic chiasma, and optic nerve. The pGTVnd was the GTVnd with an expansion of 3 mm. CTV_1_ covered the high-risk clinical target volume, and CTV_2_ included the low-risk clinical target volume. Each CTV was automatically expanded in order to generate the corresponding planning target volume (PTV) with an isotropic 3 mm margin, while assuring that the edge of the distribution was at least 2 mm from the skin. The contour of parotid glands referred to the standard of van de Water et al. [15].

Treatment planning was made on the TomoTherapy Hi-Art 2.2.4.1 workstation. Treatment was delivered in 33 fractions to the primary tumor and metastatic nodes (pGTVnx and pGTVnd) for a total of 70 Gy, while the PTV1 and PTV2 received 60 Gy and 56 Gy, respectively. The following dose-volume constraints for OARs were utilized: brainstem *D*_max_ < 54 Gy, lens *D*_max_ < 5 Gy, optic nerve *D*_max_ < 54 Gy, spinal cord *D*_max_ < 45 Gy, temporomandibular joint *D*_max_ < 60 Gy, inner ear *D*_max_ < 60 Gy, parotid gland *V*_30_ < 50%, oral cavity *V*_40_ < 30%, and larynx-oesophagus-trachea *V*_40_ < 30%.

### 2.3. Anatomical and Dosimetric Measurements of the Parotid Gland

HT's adaptive software calculated the volume and actual dose distribution according to the pretreatment MVCT scanning [14]. The MVCT images of the first fraction were collected, followed by additional 7 fractions (fractions numbers 6, 11, 16, 21, 26, 31, and 33) for a total of 8 series of images. According to previously noted setup errors, each patient's MVCT images were merged with each patient's corresponding KVCT images using the adaptive software. The same physician manually contoured the parotid glands of each patient on the MVCT images. According to the contoured images, the actual single fraction dose-volume histograms (DVHs) of the parotid gland were gained in the adaptive software. Variations in parotid volume, *D*_mean_ (mean dose), *V*_1_ (the volume receiving ≥ 1 Gy), and *D*_50_ (the dose delivered to 50% of the volume) of each fraction were extracted from the DVH gained from the Planned Adaptive application software. The ipsilateral and contralateral parotid glands were analyzed separately.

In order to assess the cumulative dosimetric differences, all data from the 8 fractions were imported as DICOM files into the computational environment for radiotherapy (CERR) [16, 17]. The cumulative delivered dose over the entire treatment course was assessed from the sum of the 8 weekly MVCT images. The total delivered dose was then evaluated on the MVCT images of the 16th fraction using the cumulated dose information from the intermediate weekly scanning images.

Anatomical parameters such as skin separation were determined on the original KVCT and MVCT images. The skin separations at the level of the odontoid processes of the C2 vertebra and the intervertebral foramens of the C1 and C4 vertebrae were used to assess the anatomical changes. Patient characteristics (e.g., age, gender, weight loss, tumor stage, primary tumor volume, total volume of metastatic nodes, initial *D*_mean_, and initial parotid volume) were collected and analyzed.

### 2.4. Statistical Analysis

Statistical analyses were performed using repeated measures analysis of variance, Pearson correlation calculations, logistic multivariate regression analysis, and receiver operating characteristic (ROC) analysis by SPSS 17.0 (SPSS Inc., Chicago, IL, USA). To determine the effectiveness of replanning cut-off values in ROC analysis, the consistency test was performed. All statistical tests were performed two-sided and a *p* value of <0.05 was considered to indicate statistical significance.

## 3. Results

### 3.1. Anatomical Changes

The average weight loss rate during radiation therapy was 10.66% (range, 3–21%). Interweekly weight variations had statistical differences. Significant changes in skin separations between the first and last fractions at the level of the odontoid processes of C2 and that of the intervertebral foramens of the C1 and C4 vertebrae were observed. Separations at the C1, C2, and C4 levels averaged −11.05%, −11.23%, and −13.29%, respectively. Reductions in skin separations were smooth during the course of radiation therapy and no plateau was found. Weight loss rates had a moderate-to-strong correlation with reductions in skin separation at the C1, C2, and C4 levels (*r* = 0.568, *p* = 0.000; *r* = 0.441, *p* = 0.017; *r* = 0.480, *p* = 0.010; resp.).

In our cohort, the data of 100 parotid glands were evaluated. The median absolute value and percentage of parotid volume reduction were 19.51 cm^3^ (range, 6.58–40.26 cm^3^) and 35% (range, 6.80–69.44%), respectively. The median parotid volume reduction was 1.07%/d of the initial volume. No differences were found between the ipsilateral and the contralateral parotid glands.

### 3.2. Dosimetric Findings

Parotid dosimetric parameters of the 8 fractions during radiation therapy included *D*_mean_, *D*_50_, and *V*_1_. The increasing rates of parotid *D*_mean_, *V*_1_, and *D*_50_ were 22.13%, 39.42%, and 48.45%, respectively. The variation trend of these dosimetric parameters between the ipsilateral and contralateral parotids was almost the same. The total cumulative parotid dose was also estimated. The initial average *D*_mean_ of 100 parotid glands was 30.28 Gy, while the cumulative average *D*_mean_ at the end of radiation therapy, as estimated by CERR, was 33.8 Gy, an increase of 11.38%. It is worth noting that increases in parotid dose were very individual and varied, as shown by the fact that cumulative parotid doses ranged from −1.51% to 30.57%.

### 3.3. Factors Affecting Parotid Volumetric and Dosimetric Changes

A summary of the relevant potential predictive factors for parotid volumetric and dosimetric changes is given in Table 2. Single-factor repetitive measurement analysis was performed. The most consistent predictive factor was the variation rate of skin separation. Reductions in skin separation at the C1 and C2 levels predicted a decrease in parotid volume and an increase in delivered dose to the parotid glands (*p* = 0.000 and *p* < 0.05, resp.). Weight loss rate was another predictive factor for volumetric and dosimetric changes (including the *D*_mean_, *V*_1_, and *D*_50_ of each of the 8 fractions) in the parotid glands (*p* < 0.05). The N stage and initial parotid volume affected the variation in parotid volume during radiation therapy (*p* = 0.044 and *p* = 0.000, resp.). Different treatment modalities affected the variations of *V*_1_ and *D*_50_ (*p* = 0.031 and *p* = 0.039, resp.); patients who received concurrent chemoradiotherapy had larger variations of *V*_1_ and *D*_50_. The initial parotid *D*_mean_ affected the variation of *D*_50_ (*p* = 0.002). Age, T stage, primary tumor volume, volume of metastatic nodes, and area of metastatic nodes did not affect parotid volumetric and dosimetric parameters.

### 3.4. Possible Prognostic Factors of Replanning

We wondered whether clinical characteristics and some externally measurable parameters, including anatomical changes, could predict the necessity of replanning. In our research, replanning was thus based on the anatomical and dosimetric parameters of the parotid gland. At the end of radiation therapy, if the cumulative actual parotid dose was more than 10% of the initial planning dose, replanning was considered to be necessary. Multivariate logistic regression analysis indicated that initial parotid volume, initial parotid *D*_mean_, and weight loss rate were significant predictive parameters for the increase of actual parotid dose (*p* < 0.05). In other words, these 3 parameters were possible prognostic factors for parotid protection-based replanning.

### 3.5. Candidate Cut-Off Values of Replanning

ROC curves were used to establish replanning cut-off values. The 3 parameters (initial parotid volume, initial parotid *D*_mean_, and weight loss rate) that were filtered from logistic analysis were used in ROC analysis. The area under the curve (AUC) and cut-off values of these parameters are shown in Table 3. To predict the necessity of replanning during treatment, weight loss rates at the 11th, 16th, and 21st fractions were used in ROC analysis. The AUC and cut-off values are shown in Table 3.

When these 3 parameters were used independently, they had weak predictive power for replanning (0.5 < AUC < 0.7) (see Table 4). To improve their predictive power, we performed a multivariate consistency check. These parameters were combined together in two ways: first, 2 of the 3 parameters reached the cut-off values; second, all 3 parameters reached the cut-off values. The results of the multivariate consistency check showed that when 2 of the 3 parameters reached the cut-off values, their predictive power was better than another combination modality (Table 5). In other words, at the 11th, 16th, and 21st fraction, when 2 of the 3 parameters including initial parotid volume, initial parotid *D*_mean_, and weight loss rate reach their cut-off values, the possibility of overdose in the parotid gland is high and the patient should then receive a replanning.

## 4. Discussion

In recent years there has been great interest in highly conformal radiation technologies with steep dose gradients between tumor and normal structures, such as IMRT, and their ability to reduce the side-effects of radiation therapy, including xerostomia, in head and neck cancer [18]. As parotid glands produce approximately 60% of saliva and their anatomical changes during radiation therapy may have significant dosimetric implications, parotid protection-based ART has become a hot field of study in head and neck cancer IMRT [19, 20]. In this study, we attempted to quantify the effect of anatomical changes on parotid dosimetry and to detect the factors that affect the actual dose that is delivered to the parotid gland. On the basis of these data, we screened out 3 cut-off values which could predict the need for replanning in parotid protection-based ART in NPC.

As shown by published studies, parotid volume decreases when the gland migrates into the high-dose volume during radiation therapy for NPC patients, leading to a higher actual parotid dose than was intended in the initial planning [8, 14]. In our study we observed that the actual parotid *D*_mean_ increased, as well as *V*_1_ and *D*_50_, after a single fraction. We also found that the magnitude of the dosimetric changes varied among our different patients, suggesting that not all NPC patients need a replanning. Recently, Hunter et al. [21] treated 18 oropharyngeal cancer patients. In order to calculate their cumulative delivered doses, parotid glands in cone-beam CT (CBCT) images were aligned by deformable registration. Stimulated salivary flow rates were measured before therapy and, periodically, after therapy. The outcomes suggested that, in most cases, ART was not likely to improve measurable salivary output. However, the researchers admitted that the residual setup error was still largely responsible for causing the dosimetric deviation that occurred after CBCT image guidance was used to correct the translational setup. Additionally, when researching the effect of adaptive replanning on locoregional control, Zhao et al. [22] and Chen et al. [23] argued that routine replanning was probably not necessary but still suggested that there would be a significant benefit if appropriate patients were selected.

In our study we screened for certain factors that would predict dosimetric variations in NPC patients during radiation therapy. Parameters such as age, T/N stage, tumor volume, initial parotid volume, weight loss rate, and reduction of facial skin separation were analyzed using correlation and logistic multivariate regression analyses. Weight loss rate was detected to be one of the most important parameters to correlate with the variation of actual parotid dose. Similar to our results, Hansen et al. [24] performed a retrospective chart review for 13 head and neck cancer patients who were treated with IMRT and received repeat CT imaging and replanning when weight loss became obvious during treatment. They showed that weight loss amplified the actual dose variation to normal tissue and bone (including the spinal cord, parotid glands, and mandible) and increased the *D*_mean_, *D*_50_, and *V*_26_ of the right parotid gland. They also suggested that weight loss might have a stronger impact on dosimetric changes than tumor shrinkage. Chen et al. [25] also found that weight loss caused significant dosimetric changes of targets and OARs in NPC patients treated with IMRT and believed that repeated scanning and replanning for patients with an obvious weight loss might be necessary. Our study's data also showed that weight loss during radiation therapy could forecast overdose to the parotid gland.

The timing of replanning is a controversial topic in parotid protection-based ART in head and neck cancer. Someone recommended replanning when it became obvious that a tumor had shrunk, weight loss had occurred, or skin separation had reduced [7, 11, 12, 21–23], while others believe that replanning should be performed when a specific fraction has been reached [26–28]. Our precious study found that parotid volume variation presented a linear pattern throughout IMRT of NPC realized by HT technique, and the rate of volume variation reached its peak at the 16th fraction and then decreased gradually, suggesting that replanning is appropriate in the fourth week [14]. In this study, we raised the specific criteria coupled with timing of replanning for parotid protection-based ART in NPC. The criteria consisted of 3 parameters: (1) an initial parotid volume of > 52.80 cm^3^; (2) an initial parotid *D*_mean_ of > 32.04 Gy; (3) a weight loss rate at the 11th fraction of > 2.3%, a weight loss rate at the 16th fraction of > 3.6%, or a weight loss rate at the 21st fraction of > 4.4%. If the patient reached 2 of these 3 parameters, the parotid gland would likely to be overdosed and a replanning was recommended at the current fraction of radiation therapy. Recently, Brown et al. [29] analyzed 110 patients with oropharyngeal squamous cell carcinoma and NPC. Patient demographics and tumor characteristics were compared between patients who were replanned and those that were not. Nodal disease stage, pretreatment of largest involved node size, diagnosis, and initial weight were identified as being significant for inclusion in the predictive model and ART risk profiles. However, among the 110 patients, only 5 had replanning, suggesting a low credibility of their model. Castelli et al. [30] estimated the parotid overdose and the xerostomia risk increase during IMRT with weekly CTs and replanning in 15 patients with locally advanced head and neck cancer. Parotid cumulated doses were estimated for the two scenarios, with or without replanning, using deformable image registration. Compared to the initial planning, a parotid overdose was observed in 59% of the parotid glands, with an average *D*_mean_ increase of 3.7 Gy. The parotid overdose increased with the tumor shrinkage and the neck thickness reduction. Weekly replanning decreased the parotid *D*_mean_ by 5.1 Gy and the absolute risk of xerostomia by 11%. However, weekly replanning consumes a large amount of medical resources and is difficult to be routinely applied.

Our criteria have at least 3 advantages. First, the parameters involved in our criteria address the combination of factors that correlate with actual volumetric and dosimetric variations in the parotid gland. We evaluated the predictive factors of these variations, screened out some parameters which could predict parotid overdose by logistic stepwise regression analysis, used ROC analysis to obtain the cut-off values of these parameters, and then used single- and multiple-factor consistency tests to confirm the predictive value of these parameters in order to combine them as replanning criteria. Hansen et al. [24] carried out replanning when obvious tumor shrinkage and/or weight loss were noted but did not clarify the replanning thresholds of these 2 parameters. A similarly ambiguous replanning standard was seen in the research of Zhao et al. [22] who compared the treatment results of replanning and no replanning in NPC patients. Recently, Lee et al. [31] studied the tumor volume reduction rate (TVRR) during radiation therapy and found that TVRR was a prognosticator of locoregional disease control in patients with oropharyngeal cancer. To ensure locoregional control, they suggested a few therapeutic modifications that were based on TVRR. Second, our parameters reflect patients' individual characteristics. As the initial parotid volume reflects the condition of the parotid gland before treatment, the initial parotid *D*_mean_ reflects how initial planning and weight loss rate correlate with the variation degree of parotid anatomical and dosimetric changes during radiation therapy. Our criteria are more comprehensive than those which paid more attention to anatomical changes but less attention to the initial conditions of the parotid gland. Initial conditions of the parotid gland, such as its initial volume and *D*_mean_, have since been confirmed to relate to the variation of parotid dose [32]. Fiorentino et al. [26] used CBCT images to analyze the parotid volume of 10 patients with head and neck cancer during radiation therapy and suggested that replanning should be performed during the third week. However, the individual conditions in our study patients make it clear that replanning at the same time point is not suitable for every patient. Third, the parameters of our criteria are easy to measure, even without repeated CT scanning during treatment. Specifically, the initial volume and *D*_mean_ of the parotid gland can be evaluated from simulation CT images and initial planning and weight loss rate can be measured easily during treatment, unlike other parameters such as facial skin separation which is only measurable by repeated CT imaging during treatment.

However, taking into account the complexity of ART, the cut-off values raised in our study have some limitations. First, these cut-off values are based on the possibility of an overdose to the parotid gland and can thus only be used to protect the parotid gland. They cannot be used to protect the other OARs or correct for an underdose to a tumor target which may not be correlated with volumetric changes. The study of Yan et al. [33] showed that GTV volume reduction was negatively correlated with the apparent diffusion coefficient (ADC) values of pretreatment tumors but not pretreatment tumor volume, and CTV volume reduction was correlated with pretreatment body mass index (BMI). Second, delineation of the parotid gland affects the use of these cut-off values. For example, when the deep lobe of the parotid gland is not contoured, our criteria will not be suitable for replanning prediction. Third, deformable registration was not used in this study. Deformable registration is being increasingly used in ART research, not only in anatomical registration but also in dose calculation [21, 30, 34–36]. We are developing home-made deformable registration software and will compare its results with those from this study. Fourth, if the initial parotid volume and *D*_mean_ are all above the cut-off values, the patient is suitable for replanning, though it is not clear when that should happen.

## 5. Conclusions

During the IMRT of NPC, the actual volume and dose of the parotid gland vary significantly in some patients. The initial volume and mean dose of the parotid gland and body weight loss rate are the most significant predictors for these variations. Replanning is suggested if these parameters reach the cut-off values recommended in this paper.

## Figures and Tables

**Table 1:** Patient characteristics.

Characteristics	Number of patients	Percent (%)
Gender		
Male	40	80
Female	10	20
Age	11–80 years (median, 44 years)	
UICC stage (2002)		
T		
1	13	26
2	16	32
3	13	26
4	8	16
N		
0	18	36
1	15	30
2	15	30
3	2	4
M		
0	50	100
Treatment method		
RT	20	40
CCRT	13	26
ICT + CCRT	17	34
Primary tumor volume	37.54 ± 25.23 (4.36–118.00) cm^3^	
Volume of metastatic nodes	13.15 ± 23.18 (0–133.35) cm^3^	
Weight loss rate at the end of RT	10.80 ± 4.12%	

RT: radiation therapy; CCRT: concurrent chemoradiotherapy; ICT: induced chemotherapy.

**Table 2:** Correlations of factors with parotid (actual) volume and dose.

Factor	Volume (*p* value)	*D*_mean_ (*p* value)	*V*_1_ (*p* value)	*D*_50_ (*p* value)
Age	0.683	0.858	0.846	0.743
T stage	0.690	0.862	0.883	0.716
N stage	0.044	0.439	0.607	0.413
Volume of primary tumor	0.712	0.422	0.689	0.093
Volume of metastatic nodes	0.086	0.463	0.521	0.308
Treatment method	0.061	0.059	**0.031**	**0.039**
Initial parotid volume	**0.000**	0.205	0.241	0.254
Initial parotid *D*_mean_	0.549	0.226	0.286	**0.002**
Weight loss rate	**0.036**	**0.000**	**0.004**	**0.014**
Reduction of skin separation at C1 level	**0.000**	**0.000**	**0.000**	**0.000**
Reduction of skin separation at C2 level	**0.010**	**0.000**	**0.000**	**0.013**
Reduction of skin separation at C4 level	**0.042**	0.090	0.110	0.271

**Table 3:** AUC and cut-off values in ROC analysis.

Parameter	AUC value	Cut-off value
Initial parotid volume	0.570	52.80 cm^3^
Initial parotid *D*_mean_	0.566	32.04 Gy
Weight loss rate	0.568	12.24%
Weight loss rate at 11th fraction	0.662	2.30%
Weight loss rate at 16th fraction	0.597	3.60%
Weight loss rate at 21st fraction	0.575	4.40%

**Table 4:** Consistency check of single factors.

Parameter	Sensitivity (%)	Specificity (%)	Positive predictive value (%)	Negative predictive value (%)	Kappa value	*p* value
Initial parotid volume > 52.80 cm^3^	73.1	41.7	57.6	58.8	0.149	0.27
Initial parotid *D*_mean_> 32.04 Gy	42.3	79.2	68.8	55.9	0.211	0.10
Weight loss rate at 11th fraction > 2.30%	50.0	66.7	61.9	55.2	0.165	0.23
Weight loss rate at 16th fraction > 3.60%	57.7	54.2	57.7	54.2	0.119	0.40
Weight loss rate at 21st fraction > 4.40%	73.1	25	56.4	41.2	0.200	0.88

**Table 5:** Consistency check of multiple factors.

Fraction number and combination	Sensitivity (%)	Specificity (%)	Positive predictive value (%)	Negative predictive value (%)	Kappa value	*p* value
Number 11						
a	69.2	66.7	69.2	66.7	0.359	0.011
b	87.5	52.4	25.9	91.7	0.143	0.155
Number 16						
a	66.7	65.2	69.2	62.5	0.320	0.025
b	77.8	53.7	26.9	91.7	0.181	0.087
Number 21						
a	84.6	54.2	66.7	76.5	0.392	0.004
b	44.0	95.7	91.7	61.1	0.388	0.002

a: two of the 3 parameters (initial parotid volume, initial parotid *D*_mean_, and weight loss rate) reached their cut-off values.

b: all the 3 parameters reached their cut-off values.

## References

[1] Teo P. M. L., Leung S. F., Tung S. Y. (2006). Dose-response relationship of nasopharyngeal carcinoma above conventional tumoricidal level: a study by the Hong Kong nasopharyngeal carcinoma study group (HKNPCSG). *Radiotherapy & Oncology*.

[2] Lee N., Harris J., Garden A. S. (2009). Intensity-modulated radiation therapy with or without chemotherapy for nasopharyngeal carcinoma: radiation therapy oncology group phase II trial 0225. *Journal of Clinical Oncology*.

[3] Lin S., Lu J. J., Han L., Chen Q., Pan J. (2010). Sequential chemotherapy and intensity-modulated radiation therapy in the management of locoregionally advanced nasopharyngeal carcinoma: experience of 370 consecutive cases. *BMC Cancer*.

[4] Su S.-F., Han F., Zhao C. (2012). Long-term outcomes of early-stage nasopharyngeal carcinoma patients treated with intensity-modulated radiotherapy alone. *International Journal of Radiation Oncology Biology Physics*.

[5] Peng G., Wang T., Yang K.-Y. (2012). A prospective, randomized study comparing outcomes and toxicities of intensity-modulated radiotherapy vs. conventional two-dimensional radiotherapy for the treatment of nasopharyngeal carcinoma. *Radiotherapy & Oncology*.

[6] Barker J. L., Garden A. S., Ang K. K. (2004). Quantification of volumetric and geometric changes occurring during fractionated radiotherapy for head-and-neck cancer using an integrated CT/linear accelerator system. *International Journal of Radiation Oncology Biology Physics*.

[7] Capelle L., Mackenzie M., Field C., Parliament M., Ghosh S., Scrimger R. (2012). Adaptive radiotherapy using helical tomotherapy for head and neck cancer in definitive and postoperative settings: initial results. *Clinical Oncology*.

[8] Han C., Chen Y.-J., Liu A., Schultheiss T. E., Wong J. Y. C. (2008). Actual dose variation of parotid glands and spinal cord for nasopharyngeal cancer patients during radiotherapy. *International Journal of Radiation Oncology Biology Physics*.

[9] Nishi T., Nishimura Y., Shibata T., Tamura M., Nishigaito N., Okumura M. (2013). Volume and dosimetric changes and initial clinical experience of a two-step adaptive intensity modulated radiation therapy (IMRT) scheme for head and neck cancer. *Radiotherapy & Oncology*.

[10] Wang R.-H., Zhang S.-X., Zhou L.-H. (2014). Volume and dosimetric variations during two-phase adaptive intensity-modulated radiotherapy for locally advanced nasopharyngeal carcinoma. *Bio-Medical Materials and Engineering*.

[11] Schwartz D. L., Garden A. S., Thomas J. (2012). Adaptive radiotherapy for head-and-neck cancer: initial clinical outcomes from a prospective trial. *International Journal of Radiation Oncology Biology Physics*.

[12] Schwartz D. L., Garden A. S., Shah S. J. (2013). Adaptive radiotherapy for head and neck cancer—dosimetric results from a prospective clinical trial. *Radiotherapy & Oncology*.

[13] Langen K. M., Meeks S. L., Poole D. O. (2005). The use of megavoltage CT (MVCT) images for dose recomputations. *Physics in Medicine and Biology*.

[14] Ren G., Xu S. P., Du L. (2015). Actual anatomical and dosimetric changes of parotid glands in nasopharyngeal carcinoma patients during intensity modulated radiation therapy. *BioMed Research International*.

[15] van de Water T. A., Bijl H. P., Westerlaan H. E., Langendijk J. A. (2009). Delineation guidelines for organs at risk involved in radiation-induced salivary dysfunction and xerostomia. *Radiotherapy & Oncology*.

[16] Deasy J. O., Blanco A. I., Clark V. H. (2003). CERR: a computational environment for radiotherapy research. *Medical Physics*.

[17] Duma M. N., Kampfer S., Schuster T., Winkler C., Geinitz H. (2012). Adaptive radiotherapy for soft tissue changes during helical tomotherapy for head and neck cancer. *Strahlentherapie und Onkologie*.

[18] Li Y., Taylor J. M. G., Ten Haken R. K., Eisbruch A. (2007). The impact of dose on parotid salivary recovery in head and neck cancer patients treated with radiation therapy. *International Journal of Radiation Oncology Biology Physics*.

[19] Bhide S. A., Davies M., Burke K. (2010). Weekly volume and dosimetric changes during chemoradiotherapy with intensity-modulated radiation therapy for head and neck cancer: a prospective observational study. *International Journal of Radiation Oncology Biology Physics*.

[20] Ahn P. H., Chen C.-C., Ahn A. I. (2011). Adaptive planning in intensity-modulated radiation therapy for head and neck cancers: single-institution experience and clinical implications. *International Journal of Radiation Oncology Biology Physics*.

[21] Hunter K. U., Fernandes L. L., Vineberg K. A. (2013). Parotid glands dose-effect relationships based on their actually delivered doses: implications for adaptive replanning in radiation therapy of head-and-neck cancer. *International Journal of Radiation Oncology Biology Physics*.

[22] Zhao L., Wan Q., Zhou Y., Deng X., Xie C., Wu S. (2011). The role of replanning in fractionated intensity modulated radiotherapy for nasopharyngeal carcinoma. *Radiotherapy and Oncology*.

[23] Chen A. M., Daly M. E., Cui J., Mathai M., Benedict S., Purdy J. A. (2014). Clinical outcomes among patients with head and neck cancer treated by intensity-modulated radiotherapy with and without adaptive replanning. *Head & Neck*.

[24] Hansen E. K., Bucci M. K., Quivey J. M., Weinberg V., Xia P. (2006). Repeat CT imaging and replanning during the course of IMRT for head-and-neck cancer. *International Journal of Radiation Oncology Biology Physics*.

[25] Chen C., Fei Z., Chen L., Bai P., Lin X., Pan J. (2014). Will weight loss cause significant dosimetric changes of target volumes and organs at risk in nasopharyngeal carcinoma treated with intensity-modulated radiation therapy?. *Medical Dosimetry*.

[26] Fiorentino A., Caivano R., Metallo V. (2012). Parotid gland volumetric changes during intensity-modulated radiotherapy in head and neck cancer. *The British Journal of Radiology*.

[27] Fung W. W. K., Wu V. W. C., Teo P. M. L. (2014). Developing an adaptive radiation therapy strategy for nasopharyngeal carcinoma. *Journal of Radiation Research*.

[28] Brock K., Lee C., Samuels S. (2015). TU-AB-303-05: clinical guidelines for determining when an adaptive replan may be warranted for head and neck patients. *Medical Physics*.

[29] Brown E., Owen R., Harden F. (2015). Predicting the need for adaptive radiotherapy in head and neck cancer. *Radiotherapy & Oncology*.

[30] Castelli J., Simon A., Louvel G. (2015). Impact of head and neck cancer adaptive radiotherapy to spare the parotid glands and decrease the risk of xerostomia. *Radiation Oncology*.

[31] Lee H., Ahn Y. C., Oh D., Nam H., Kim Y. I., Park S. Y. (2014). Tumor volume reduction rate measured during adaptive definitive radiation therapy as a potential prognosticator of locoregional control in patients with oropharyngeal cancer. *Head & Neck*.

[32] Broggi S., Fiorino C., Dell'Oca I. (2010). A two-variable linear model of parotid shrinkage during IMRT for head and neck cancer. *Radiotherapy & Oncology*.

[33] Yan D., Yan S., Wang Q., Liao X., Lu Z., Wang Y. (2013). Predictors for replanning in loco-regionally advanced nasopharyngeal carcinoma patients undergoing intensity-modulated radiation therapy: a prospective observational study. *BMC Cancer*.

[34] Kumarasiri A., Siddiqui F., Liu C. (2014). Deformable image registration based automatic CT-to-CT contour propagation for head and neck adaptive radiotherapy in the routine clinical setting. *Medical Physics*.

[35] Gu X., Dong B., Wang J. (2013). A contour-guided deformable image registration algorithm for adaptive radiotherapy. *Physics in Medicine and Biology*.

[36] Lu J., Ma Y., Chen J. (2014). Assessment of anatomical and dosimetric changes by a deformable registration method during the course of intensity-modulated radiotherapy for nasopharyngeal carcinoma. *Journal of Radiation Research*.

